# Fat and Iron Don’t Mix

**DOI:** 10.20900/immunometab20200034

**Published:** 2020-10-08

**Authors:** Magdalene K. Ameka, Alyssa H. Hasty

**Affiliations:** Department of Molecular Physiology and Biophysics, Vanderbilt University, 23rd Ave South and Pierce, Nashville 37232-0615 USA

**Keywords:** adipose tissue homeostasis, adipose tissue macrophages, iron, insulin resistance

## Abstract

Low-grade chronic adipose tissue (AT) inflammation is now recognized as a pivotal driver of the multi-organ dysfunction associated with obesity-related complications; and adipose tissue macrophages (ATMs) are key to the development of this inflammatory milieu. Along with their role in immunosurveillance, ATMs are central regulators of AT iron homeostasis. Under optimal conditions, ATMs maintain a proper homeostatic balance of iron in adipocytes; however, during obesity, this relationship is altered, and iron is repartitioned into adipocytes as opposed to ATMs. This adipocyte iron overload leads to systemic IR and the mechanism for these effects is still under investigation. Here, we comment on the most recent findings addressing the interplay between adipocyte and ATM iron handling, and metabolic dysfunction.

A salient inflection point in the progression of multiple metabolic diseases is an infiltration of immune cells and a shift to a pro-inflammatory signature in key metabolic organs including adipose tissue (AT). In obese AT, these phenotypic changes contribute to a persistent low-grade inflammatory milieu and general AT dysfunction [1]. Macrophages (Mϕs) represent a majority of the infiltrating pro-inflammatory immune cells and their altered crosstalk in the AT environment leads to further dysfunction, even in distal tissues [2]. By producing chemokines and inflammatory cytokines, these Mϕs perpetuate a cycle of leukocyte infiltration, inflammation and insulin resistance in obese AT. The local insulin resistant state in AT leads to increased lipolysis and production of toxic fatty acid intermediates which attenuate insulin’s action in other metabolic organs [3]. Cognizant that Mϕ-driven AT inflammation is a nascent event in the cascade of systemic insulin resistance, there has been burgeoning interest in unraveling the interplay between Mϕs and adipocytes. While most of the early work in this field focused on the damaging inflammatory actions of AT Mϕs (ATMs), recent studies suggest that ATMs may have important non-inflammatory roles in both lean and obese conditions and that these pro and anti-inflammatory effects of obesogenic ATMs are not always detrimental to AT health [4-9]. For example, inflammatory Mϕs are critical for AT development [8,10] and the removal of cellular waste after obesity-induced adipocyte death [11]. Another example is that anti-inflammatory Mϕs have been associated with insulin resistance in one study of human ATMs [12]. These findings underscore that a more nuanced understanding of the nature of the role of Mϕs in AT is warranted. In line with this idea, we have been studying another aspect of ATM function, iron regulation, which recent studies pinpoint as being critical for AT homeostasis. In this Viewpoint, we will discuss the current knowledge of iron-ATM-adipocyte relationships and their effects on metabolism, with the end goal of highlighting how this axis may provide novel avenues to treat metabolic disease. For the sake of clarity, we distinguish between what is known about AT as a whole organ with many different cell types versus the distinct cells, adipocytes and ATMs.

Little is known about the effects of iron deficiency on normal AT biology; however, a state of systemic anemia that accompanies chronic inflammatory conditions like obesity, has been described. Interestingly, this phenotype is driven by adaptations that cause localized tissue iron sequestration while depleting systemic stores of iron [13]. The relationship between iron overload and metabolic disease in humans is acknowledged and is reciprocal. Metabolic syndrome and non-alcoholic fatty liver disease are significantly associated with iron overload in metabolic tissues [14] and 10% of patients with non-genetic forms of general tissue iron overload also have type 2 diabetes mellitus [15]. Supporting a direct role of iron in metabolic disease risk, iron chelation improves insulin sensitivity in humans [16] and in mice with insulin resistance [17,18]. Additionally, glycation of hemoglobin, a protein with the highest stores of functional iron is a cornerstone of determining long-term glycemic control in humans. Therefore, iron homeostasis may be a vital underappreciated node of metabolic homeostasis in humans. While we focus here on the effects in AT, we underscore that iron homeostasis is critical, such that iron deficiency and overload are generally detrimental in all tissues.

Mϕs are ubiquitous in all tissues [19] and are the regulators of central iron homeostasis [20,21]. Systemically, specialized Mϕs in the spleen and liver recycle iron from millions of replete erythrocytes daily. This process supplies most of the body’s iron needs as dietary sources only provide about 5% of the body’s daily iron requirement [22]. Interestingly, distinct iron profiles help define Mϕ polarization phenotypes and functions. The pro-inflammatory M1 Mϕs sequester and store iron to limit microbial use of this iron for their own replication and virulence while the anti-inflammatory M2 Mϕs cycle iron and are able to supply or take excess iron away from cells, thus acting as local “ferrostats” [23,24]. There has been a growing appreciation in the field that the M1/M2 classification scheme does not accurately represent ATMs in obesity [25,26]. Different studies classify these macrophages along a spectrum of terms including M1/M2 hybrid [25], foamy macrophages [27], lipid associated macrophages [28] or metabolically activated macrophages [29]. As such, we are just beginning to understand how these differentially polarized states affect specialized ATM functions in obesity. We are primarily interested in understanding how obesity affects ATM iron handling roles and how ATM iron handling impacts nearby adipocytes. Recently, we reviewed the importance of tissue resident Mϕs in tuning local iron equilibria in different systems [23], including AT. In this Viewpoint, we discuss how AT iron homeostasis, based on the iron handling relationship between adipocytes and ATMs, influences AT function and metabolic homeostasis.

Iron is required in AT for normal homeostatic function. It is required for adipocyte growth in just the right concentrations so that too much or too little iron impedes adipogenesis. Under lean conditions, loss of transferrin [30] and lactoferrin [31], soluble iron-carrying proteins that are highly expressed in ATMs, recapitulated the anti-adipogenic effects of in vitro chelation [30]. In vitro chelation of iron in already obese mice ameliorated oxidative stress and inflammation and improved markers of insulin action [17,18]. In these studies, these effects were attributed to loss of iron in adipocytes; however, because ATMs were not assessed, their involvement in these improvements cannot be conclusively ruled out. In another study, the impact of anemia on obesity-related metabolic phenotypes was tested [32]. Mice that were anemic were more obese than normo-ferric mice on the same high fat diet, and this was attributed to decreased AT browning. The obese anemic mice were intolerant to acute cold and also showed reduced expression of uncoupling protein 1 (UCP1), CytC and reduced beiging in the inguinal fat [32]. Given the primacy of their role in cellular iron trafficking, it is tempting to speculate that the effects in the obese anemic mice were at least in some part due to defective iron handling in ATMs. These findings suggest that systemic iron depletion impinges upon normal AT function and may lead to widespread energetic imbalance and metabolic dysfunction.

Iron excess in AT is particularly detrimental considering the lipid-rich environment. One mechanism for iron-related dysfunction in AT is the propensity of free reactive iron to catalyze Fenton and Harber Weiss reactions with lipid intermediates leading to the generation of reactive oxygen species that limit tissue function and cause iron-mediated cell death [33,34]. AT hypoxia is a major contributor to general maladaptive AT dysfunction with obesity and AT iron overload as part of its etiology [35,36]. For example, in KK/HIJ mice, a polygenic model of obesity, a subgroup spontaneously develop adipocyte iron overload in their epididymal AT (eAT) of up to a 100-fold excess, that was revealed by a characteristic grossly bronze appearance of the tissue. Interestingly, the same subgroup of mice did not show any changes in iron in their brown or subcutaneous AT, both depots with more iron-rich mitochondria and higher UCP-1 expression than the eAT. This preferential iron deposition was accompanied by significant eAT hypoxia, remodeling, tissue fibrosis and local insulin resistance [37]. These data suggest that the effects of iron overload can indeed be further localized to specific AT beds; however, whether these patterns of dysregulated iron homoeostasis and AT dysfunction exist in other models of genetic and diet induced obesity is currently unclear.

Both in vitro and in vivo studies reveal the importance of iron to adipokine production by adipocytes. Treatment of 3T3-L1 adipocytes with iron sulfate promoted insulin resistance in vitro by limiting transcription of the insulin sensitizing adipokine, adiponectin. Furthermore, excess iron was shown to drive a net decrease in adiponectin promoter activity by FOXO1’s trans-repressing activity on response elements in the adiponectin promoter [38]. In vivo, mice fed an iron-rich diet had significantly lower levels of serum adiponectin while those fed a low iron diet had significantly higher levels compared to normal chow-fed mice [38]. In humans with metabolic syndrome, serum ferritin levels were tightly associated with adiponectin levels after accounting for inflammation, BMI or previous diabetic status [38]. Furthermore, regular phlebotomy, which decreases iron stores, results in increased levels of adiponectin and improved glucose tolerance [38]. Therefore, a reduction of adiponectin may be a mechanism by which iron excess causes metabolic dysfunction in AT and systemically. In keeping with the effects of iron on adipokine production, the same group found an inverse relationship between human ferritin levels and serum leptin that was independent of BMI or inflammation. A similar in vitro and in vivo paradigm of iron excess led to a decrease in leptin expression in cultured primary and 3T3-L1 adipocytes and significant hyperphagia in hyper-ferric mice. Iron inhibited the transcription of leptin in a mechanism that required intact phosphorylated CREB at the leptin promoter and these effects were completely reversed by iron chelation or dominant negative mutation of CREB [39]. Together, these data showcase at least two independent roles for iron in regulating adipokines and their subsequent actions.

The studies, we highlight in the paragraphs above, focus on the effects of iron overload in AT—an environment with a mix of different cell types, all of which could be contributing to the cumulative AT iron flux. To study the effects of adipocyte-specific iron homeostasis on metabolism, models in which iron import and export proteins have been ablated, provide important insights. Adipocyte-targeted knock down of iron handling proteins [31,40] in vitro and in vivo are generally associated with dysfunctional elevation in inflammation and reductions in insulin signaling. For example, human adipocytes lacking lactoferrin showed increased expression of inflammatory genes [31]. Mice lacking transferrin receptor only in adipocytes are iron-deficient under lean conditions. On a high-fat diet, these mice have increased markers of AT inflammation, dysregulated lipid metabolism, mount a defective thermogenic response, and develop insulin resistance. Interestingly, these effects were thought to be regulated by HIF1α activation in beige adipocytes [40]. This is entirely in line with the observations noted above in the KK/HIJ and obese anemic mice [32,37]. Adipocyte iron overload is also a recurring theme in studies investigating the effects of iron excess on metabolic homeostasis [40-42]. In fact, the above-mentioned effects of iron in decreasing adiponectin and leptin have been replicated in mouse models of adipocyte-specific iron overload [38,39,43]. Several groups have studied adipocyte iron overload by blocking adipocyte iron export via ablation of ferroportin (Fpn), the main (if not only) known iron export protein [44,45] in lean [38] and obese [43] mice. In the lean animals, adipocyte Fpn deletion using a mouse model of AP2 *Cre* and Fpn^fl/fl^ caused systemic glucose intolerance by downregulating the production of adiponectin through decreased acetylation of FOXO1 and a co-repressive effect by FOXO1’s binding to a PPARγ response element [38]. In the more adipocyte-specific adiponectin *Cre* transgenic line, a similar design was employed to target adipocyte Fpn. This group found that ablating adipocyte iron overload in obese mice had no effect on insulin sensitivity or adiponectin levels [43]. The reason for the different results could be due to a difference in mouse strains, housing conditions, length of time on diet, but may also be due to the efficiency and specificity of gene editing by the cell-specific promoter *Cre* constructs. Given that AP2 can target other cell types including Mϕs, and that in the referring study, deletion of Fpn in ATMs was not confirmed, it is possible that the loss of Fpn in ATMs was responsible for the reduction in adiponectin and glucose intolerance. Indeed, we have shown that high iron regimens alone are enough to shift the phenotypes of resident ATMs [46] suggesting that manipulating ATM iron load may be a viable mechanism to influence AT health. Given that both adipocyte iron deficiency and excess lead to metabolic dysfunction, these studies underscore that an optimal adipocyte iron equilibrium is crucial to systemic metabolic homeostasis.

ATMs may be part of the etiology of adipocyte iron overload and our own studies in resident ATMs support this hypothesis. In keeping with an anti-inflammatory state, ATMs putatively fine-tune iron concentrations in AT; however, even in the lean state, there is a dichotomy in tissue resident ATMs based on their ability to handle iron. We have shown that lean AT has a high iron recycling Mϕ population (MFe^hi^) and a low iron recycling Mϕ population (MFe^lo^)—the MFe^hi^ being important for buffering tissue iron concentrations [6]. Interestingly, our data suggest that in conditions of high iron, the relative proportion of MFe^hi^ Mϕs increases mostly through reconfiguration of MFe^lo^ ATMs to an MFe^hi^ signature; however, net ATM numbers remain the same. This serves not only to protect adipocytes from harmful effects of iron overload, but also maintain an overall anti-inflammatory tone in AT in conditions that would normally be toxic. Indeed, indicators of adipocyte dysfunction and inflammation, adiponectin and Il6 gene expression remained unchanged, even in the face of a high iron diet [46]. With obesity, MFe^hi^ ATM numbers per gram of tissue remain the same while the number of MFe^lo^ ATMs increase because of an influx of circulating monocytes that phenocopy MFe^lo^ resident ATMs. There is a marked loss in expression of iron handling genes in both MFe^hi^ and MFe^lo^ ATMs and the iron appears to be repartitioned away from MFe^hi^ ATMs. This net loss in AT iron buffering is accompanied by a 4-fold increase in adipocyte iron stores and a depletion of liver iron stores [6]. This reapportioning of iron in the liver and visceral AT with obesity and insulin resistance has also been observed by another group [42]. These results suggest that MFe^hi^ ATMs are crucial for the maintenance of optimal AT iron concentrations and when that function is impeded, AT health is jeopardized. Indeed, a recent profiling of metabolically activated human monocyte-derived Mϕs, which are maintained in obesogenic conditions, found that these Mϕs show an unexpected reduction in iron import proteins [29], suggesting that during obesity, human ATMs are refractory to the effects of environmental iron excess. Interestingly, obesogenic ATMs also showed increased lysosomal biogenesis [4]. It has been postulated that this event is initiated by AT-derived factors, which stimulate ATMs to take up excess lipid during the early onset of obesity in a bid to protect other cells from lipotoxicity. With prolonged obesity, the lipid catabolism in obesogenic ATMs increases whole AT lipolysis and insulin resistance by generating toxic signaling lipid moieties [4]. Interestingly, transferrin has been found to enhance the lipolytic effects of serum on adipocytes [47]; linking adipocyte iron homeostasis to lipolysis. Furthermore, palmitate administration has been reported to led to a state of functional iron deficiency [30]. We find it fascinating that at the same time that obese ATMs increase their lipid uptake and lipid catabolism, they seemingly lose their ability to buffer iron. We suggest that these events may not be mutually exclusive. In summary, studies presented here show that the responsiveness of MFe^hi^ ATMs and adipocytes to iron changes with obesity; while MFe^hi^ ATMs lose their ability to react to iron excess, adipocytes take up and store the excess iron. These studies support the finding that loss of ATM iron handling may cause insulin resistance in several ways, including increased ROS production due to reactivity of lipids and free iron [48], loss of insulin sensitivity through downregulation of adiponectin [38] and leptin [39], and aberrant lysosomal biogenesis in ATMs [4].

While there is a clear association between iron handling and the effectiveness of M1 and M2 Mϕs to carry out their immunologic functions, we can only speculate on this association in ATMs given our recent data highlighted above. In high iron conditions, MFe^hi^ Mϕs maintain a low inflammatory profile and this may tune the inflammation in AT during iron overload [46]. However, these studies were conducted in the absence of overt obesity and we have shown that obesity drives a downregulation of iron handling in these MFe^hi^ Mϕs [6]. Additionally, MFe^hi^ Mϕs represent a specialized ATM population that is resident to AT. During obesity, most of the Mϕs driving AT dysfunction infiltrate the tissue and are hematopoietically-derived. Given that obese AT is iron-overloaded, this suggests that in lean conditions, where resident M2-like MFe^hi^ Mϕs predominate, the iron handling capacity of these Mϕs is intact and adipocytes are not overloaded. During obesity, infiltrating obese ATMs may also have reduced iron handling capacity, all the while remaining inflammatory, leading to deleterious adipocyte inflammation and iron overload.

In conclusion, these data delineate a possible causative role of AT iron homeostasis in the development of insulin resistance and suggest that ATM iron handling may be crucial for proper AT iron homeostasis. These studies support the growing observation that ATMs have other inflammatory and non-inflammatory roles in exacerbating or mitigating the effects of increased adiposity.
